# Phytochemical Profile, Antioxidant Activity, and Anti‐Neuroinflammatory Effects of a Novel Dry Extract of *Libidibia ferrea* in Microglial Cells

**DOI:** 10.1002/cbdv.71708

**Published:** 2026-09-27

**Authors:** Francisco Cirineu das Chagas Neto, Kirley Marques Canuto, Arcelina Pacheco Cunha, Nágila Maria Pontes Silva Ricardo, Ana Bruna De Araújo, Francisco Vinícius Clemente Serra Azul, Paulo R. V. Ribeiro, Patrícia Maria Pontes Thé, Antônia Torres Ávila Pimenta, Luzia Kalyne Almeida Moreira Leal

**Affiliations:** ^1^ Center for Pharmaceutical and Cosmetic Studies Faculty of Pharmacy, Dentistry and Nursing Federal University of Ceará Fortaleza Ceará Brasil; ^2^ Embrapa Tropical Agroindustry Fortaleza Ceará Brasil; ^3^ Department of Organic Chemistry Federal University of Ceará Fortaleza Ceará Brasil

**Keywords:** anti‐neuroinflammatory activity, inflammatory mediators, *Libidibia ferrea*, methyl gallate, microglial cells, polyphenols

## Abstract

*Libidibia ferrea* is traditionally medicine used in northeastern Brazil; however, studies addressing chemically characterized extracts and pharmacological properties remain limited. This study investigated the chemical composition and anti‐neuroinflammatory activity of a dry extract of *L. ferrea* (DELF) in lipopolysaccharide (LPS)‐stimulated microglial cells, as well as its possible mechanisms of action. The extract was characterized using UPLC‐ESI‐QTOF‐MS, and methyl gallate, the major compound, was quantified (38.15 µg/mg dry extract). Cytotoxicity in BV‐2 cells was evaluated by the MTT assay. Antioxidant activity was assessed using free radical scavenging assays, while nitric oxide (NO) and IL‐6 levels were determined using the Griess reaction and ELISA. iNOS expression was analyzed by western blotting. Eighteen compounds were identified, including phenolic acids, flavonoids, and hydroxy fatty acids. DELF also presented relevant mineral content. The extract (up to 100 µg/mL) and methyl gallate (up to 1.9 µg/mL) were non‐cytotoxic. DELF at 100 µg/mL reduced nitrite release by 67% in LPS‐stimulated cells and significantly reduced IL‐6 production by 55%. DELF decreased NO levels without altering iNOS expression and exhibited antioxidant activity (90.16% DPPH, 83.6% superoxide, and ∼45% hydroxyl radical scavenging at 100 µg/mL). These findings highlight DELF as a promising source of bioactive compounds with potential anti‐neuroinflammatory applications.

## Graphical abstract

A dry extract of *Libidibia ferrea* (DELF) exhibits a phenolic profile associated with antioxidant and anti‐inflammatory activities. Free radical scavenging is demonstrated against DPPH, hydroxyl, and superoxide radicals, together with reduced NO and IL‐6 production in BV2 microglial cells, highlighting DELF as a promising source of bioactive compounds.

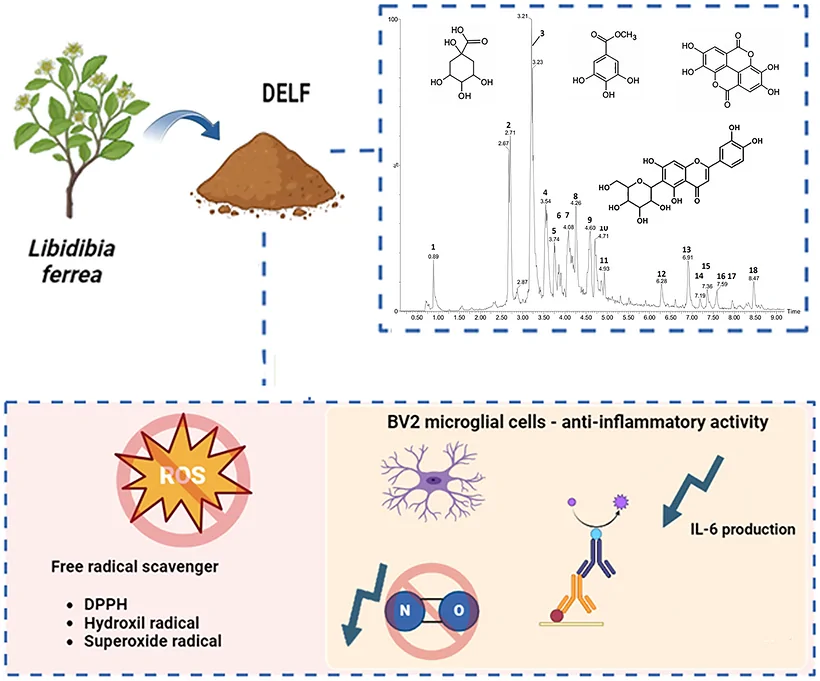

## 1 Introduction

*Libidibia ferrea* var. *glabrescens* (Benth.) L.P. Queiroz (Fabaceae family), popularly known in Northeast Brazil as “jucá,” is widely used in folk medicine and also serves as animal feed [1]. Chemical studies of *L. ferrea* allowed the identification of several secondary metabolites, including flavonoids, saponins, tannins, coumarins, sterols, and phenolic compounds in stem bark and leaves. In bark, saponins, cardiotonic glycosides, and alkaloids were found [2]. Recently, our group demonstrated the potential of galactomannan isolated from *L. ferrea* seeds as a functional food, reducing hyperglycemia and total fatty acids in diabetic rats [3].

A few studies [4, 5, 6, 7] in the literature have evaluated the nutritional and pharmacological properties of *L. ferrea*. Previous studies have demonstrated the antioxidant and anti‐inflammatory activity of the aqueous extract of *L. ferrea* leaves obtained by infusion and turboextraction [8, 9].

The use of polyphenol‐rich plants as supplements and medicines is widespread (e.g*., Camellia sinensis* and *Ginkgo biloba*). However, plant extracts can cause adverse effects, so it is essential to have chemical, pharmacological, and toxicological knowledge of species traditionally used by the population as food or medicine, such as *L. ferrea*. This is a public health concern [10].

Neuroinflammation is a critical driver in the pathogenesis of neurodegenerative disorders, with microglia playing a pivotal, dual role. Upon activation, microglia release pro‐inflammatory cytokines (e.g., TNF‐α, IL‐6) and reactive oxygen and nitrogen species (ROS/RNS), including nitric oxide (NO), inducing metabolic and oxidative stress that impairs synaptic transmission and promotes neurodegeneration [11]. Conversely, shifting microglia toward an anti‐inflammatory, neuroprotective state is a major therapeutic goal. In this context, plant‐derived polyphenols have emerged as promising candidates, as they can modulate microglial activation, suppress pro‐inflammatory pathways (e.g., NF‐κB, TLR4, and the NLRP3 inflammasome), and mitigate oxidative stress [12]. Given the close interplay between oxidative stress and microglial‐mediated neuroinflammation, investigating novel polyphenol‐rich extracts is of paramount importance.

The present study aimed to determine, for the first time, the chemical composition of a novel dry extract of *L. ferrea* leaves (DELF), obtained by a combination of homogenizer‐assisted extraction and maceration, using chromatographic analysis, and to evaluate its anti‐neuroinflammatory activity and potential mechanisms of action. Unlike previous studies that employed aqueous infusions or turboextraction [8, 9], the DELF here described is a standardized, water‐soluble powder with enhanced phenolic content, representing a novel pharmaceutical and nutraceutical formulation.

## 2 Results and Discussion

The DELF presented a total phenol content (TPC) of 260.0 ± 0.01 mg GAE/g, being part of these flavonoids (21.1 ± 0.02 mg QE/g) (Table 1). Port´s et al. [8] also evaluated the TPC of the extract from leaves of *L. ferrea* and obtained a content that was lower when compared to our extract (68.1 ± 15.93 mg GAE/g). This difference in TPC may be at least in part due to differences in extraction methods. DELF was produced by the combination of homogenizer‐assisted extraction (Ultra‐Turrax) and maceration using a methanol/water mixture as solvent, while Port's et al. [8] produced an aqueous extract of *L. ferrea* leaves by infusion. Corroborating our results, Rocchetti et al. [13], analyzing the impact of different extraction methods and solvents on the profile of phenols of *Moringa oleifera* leaves, observed that homogenizer‐assisted extraction showed the highest TPC (35.1 mg/g dry matter), followed by ultrasound‐assisted extraction (26.3 mg/g dry matter) and microwave‐assisted extraction (22.4 mg/g dry matter).

**TABLE 1:** Elemental and phenolic concentrations of dry extract of *Libidibia ferrea*.

Parameters	Concentration
Ca	169.92 ± 0.45 mg/100 g
Fe	21.65 ± 0.01 mg/100 g
Mg	231.43 ± 0.39 mg/100 g
Mn	4.38 ± 0.01 mg/100 g
Pb	Below detection limit
Zn	31.52 ± 0.02 mg/100 g
Total phenols	260.00 ± 0.01 mg GAE/g
Total flavonoids	21.10 ± 0.02 mg QE/g

*Note*: DELF showed high TPC and metal content (iron and zinc) and low flavonoid content. The analysis of metal content was performed by ICP‐OES; TPC was determined using the Folin‐Ciocalteu reagent; and flavonoid content was determined using AlCl_3_ 2%.

Abbreviations: GAE, gallic acid equivalents; QE, quercetin equivalents; TPC, total phenolic content.

DELF showed a content of TPC 65% higher than that found in the commercial extract of Camellia sinensis (157.5 mg GAE/g), while in relation to total flavonoid content, the DELF has twice as much as in the commercial extract of C. sinensis (10.0 ± 0.01 mg QE/g), a plant species whose antioxidant and anti‐neuroinflammatory properties are associated with the presence of polyphenols [14].

Phenolic compounds are among the largest families of secondary metabolites produced by plants for defense, and they have been shown to possess several properties useful for the prevention and treatment of various diseases [15]. The richness of phenols, including flavonoids, as shown by DELF, makes it a promising natural product, given the health benefits reported in the literature for these metabolic classes [16]. Thus, we characterized this material to elucidate its main constituents.

**FIGURE 1:**
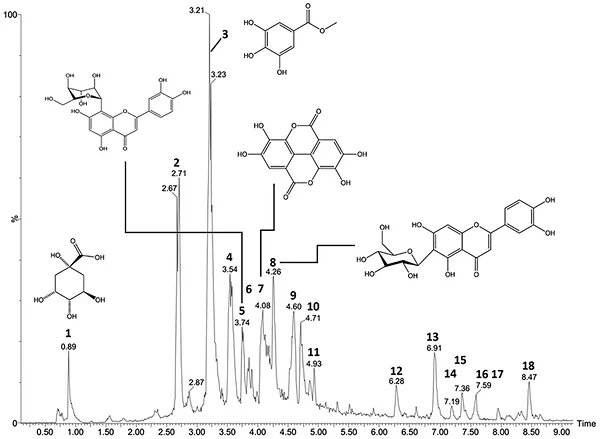
Chromatogram and chemical structures of the compounds identified in the dry extract of *L. ferrea*.

A total of 18 compounds were identified in the DELF by ultra‐performance liquid chromatography coupled to mass spectrometry. The compounds were tentatively characterized using MassLynx 4.1 software to determine molecular formulas from accurate masses (error < 5 ppm), isotopic patterns (i‐fit), and MS fragmentation patterns, and by chemotaxonomic survey. Additionally, compounds were assigned by comparison with available reference standards (Table 2). The chemical analysis of the DELF allowed the identification of polyphenols, phenolic acids (quinic acid, methyl gallate, and ellagic acid), flavonoids (orientin and isoorientin) (Figure 1), and hydroxy‐fatty acids. The major constituent of the extract is methyl gallate, which was quantified by HPLC‐PDA at a concentration of 38.15 µg/mg in the dry extract. Among the detected constituents, methyl gallate was selected as a targeted quantitative marker based on its chromatographic prominence, adequate peak resolution, availability of an authentic reference standard, and its subsequent evaluation in the biological assays. It possesses well‐established antioxidant and anti‐inflammatory activities in the literature, supporting its relevance as a bioactivity‐associated chemical marker [17, 18]; and its quantification provides a reproducible and practical parameter for quality control and batch standardization of the dry extract.

**TABLE 2:** Secondary metabolites from *Libidibia ferrea* leaves extract.

Peak no.	Rt min	[M − H]^−^ Observed	[M − H]^−^ Calculated	Product ions (MS/MS)	Molecular formula	Ppm (error)	Putative name	References
**1**	0.89	191.0558	191.0556	127.0390 (5%)	C_7_H_12_O_6_	1.0	Quinic acid^b^	
**2**	2.71	232.1195	232.1198	146.0805 (5%)	C_11_H_15_N_5_O	−1.3	Unknown	—
**3**	3.21	183.0288	183.0293	168.0015 (8%), 124.0138 (100%)	C_8_H_8_O_5_	−2.7	Methyl gallate^c^	[19]
**4**	3.56	1133.0786	1133.0802	300.9979 (5%), 169.0084 (3%)	C_45_H_34_O_35_	−1.4	Ellagic acid derivative	—
**5**	3.74	447.0933	447.0927	357.0575 (15%), 327.0485 (15%)	C_21_H_20_O_11_	1.3	Orientin^a^	[20]
**6**	3.86	1133.0801	1133.0802	300.9961 (8%), 169.0119 (5%)	C_45_H_34_O_35_	−0.1	Ellagic acid derivative	—
**7**	4.15	300.9989	300.9984	169.0134 (5%)	C_14_H_6_O_8_	1.7	Ellagic acid^b^	—
**8**	4.26	447.0927	447.0927	429.0794 (100%), 357.0584 (30%), 327.0474 (60%)	C_21_H_20_O_11_	0.0	Isoorientin^a^	[20]
**9**	4.60	583.1077	583.1088	431.0969 (50%), 269.0436 (1%), 169.0106 (58%)	C_28_H_24_O_14_	−1.9	Apigenin galloylhexoside^c^	[21]
**10**	4.71	483.0200	483.0200	300.9938 (60%), 169.0173 (5%)	C_22_H_12_O_13_	0.0	Ellagic acid derivative	—
**11**	4.93	483.0207	483.0200	300.9953 (30%), 169.0073 (5%)	C_22_H_12_O_13_	1.4	Ellagic acid derivative	—
**12**	6.28	401.1796	401.1812	373.1488 (40%), 227.1261 (38%), 145.0119 (100%)	C_19_H_30_O_9_	−4.0	Unknown	—
**13**	6.91	327.2180	327.2171	229.1413 (18%), 211.1318 (20%), 171.1018 (10%)	C_18_H_32_O_5_	2.8	Trihydroxy‐octadecadienoic acid^c^	[22]
**14**	7.19	227.1274	227.1283	183.1362 (100%), 165.1269 (15%)	C_12_H_20_O_4_	−4.0	Unknown	—
**15**	7.36	329.2324	329.2328	229.1414 (22%), 211.1315 (28%), 171.1000 (13%)	C_18_H_34_O_5_	−1.2	Trihydroxy octadecaenoic acid^c^	[22]
**16**	7.59	287.2217	287.2222	269.2026 (5%)	C_16_H_32_O_4_	−1.7	Dihydroxyhexadecanoic acid^c^	[22]
**17**	7.95	691.3539	691.3541	609.2628 (5%), 415.1458 (45%), 171.1038 (5%)	C_33_H_56_O_15_	−0.3	Unknown	—
**18**	8.47	596.2646	596.2657	—	C_23_H_46_O_17_	−1.9	Unknown	—

^a^ Identified in previous studies in the family.

^b^ Comparison with an authentic standard.

Peak 1 is a precursor ion at *m/z* 191.0504 (C7H12O6) with a product ion at *m/z* 127.0387 corresponding to the loss of two water molecules and HCOOH and was identified as quinic acid by comparing with an authentic analytical standard. Peak 3 is a precursor ion at *m/z* 183.0194 (C_8_H_8_O_5_) and fragment ions at *m/z* 168.0019 [M − H‐methyl]^−^ and 124.0138 [M − H‐methyl‐CO_2_]^−^ corresponding to methyl gallate [19]. Peak 7 showed a precursor ion at *m/z* 300.9923 (C1_4_H_6_O_8_) and a product ion at *m/z* 169.0106 (gallic acid); therefore, it was identified as ellagic acid by comparison with an authentic analytical standard. Likewise, peaks 4, 6, 10, and 11 at *m/z* 1133 (4 and 6) and *m/z* 483 (10 and 11) were identified as ellagic acid derivatives based on fragmentation patterns similar to those of ellagic acid. Both peaks 5 and 8 showed a deprotonated ion at *m/z* 447.0 (C_21_H_20_O_11_), consistent with orientin/isoorientin. The discrimination was achieved based on their fragment ions: *m/z* 429 is found only for isoorientin (peak 8) and can be used to distinguish 6‐*C*‐glycosidic from 8‐*C*‐glycosidic flavonoid [20]. Peak 9 at *m/z* 583.1088 (C_28_H_24_O_14_) exhibited losses of galloyl and hexose at *m/z* 431.0969 and 269.0436 (apigenin), respectively, and was tentatively identified as apigenin galloyl hexoside [21]. Peaks 13, 15, and 16 were tentatively identified as fatty acids. Peaks 13 and 15 showed a precursor ion at *m/z* 327.2180 (C_18_H_32_O_5_) and 329.2324 (C_18_H_34_O_5_), respectively, indicating the existence of an extra double bond for the former one by comparison to the compound previously identified in literature data. They were tentatively identified as trihydroxyoctadecadienoic acid, trihydroxyoctadecaenoic acid, and dihydroxyhexadecanoic acid, respectively [22].

The analyses of elemental contents of DELF were carried out by ICP‐OES, a multi‐elemental sensitive technique (Table 1). Minerals are essential micronutrients for body homeostasis. Zinc and magnesium participate in enzymatic reactions, calcium and manganese are fundamental for bone resistance, and iron is a component of hemoglobin. The analyses identified five minerals in DELF: Ca, Fe, Mg, Mn, and Zn. According to the dietary reference intakes (DRIs) [23], DELF contains high levels of metals, including iron (21.6 ± 0.01 mg/100 g), manganese (4.3 ± 0.01 mg/100 g), and zinc (31.5 ± 0.02 mg/100 g).

Some studies [14, 24] suggest that polyphenol‐rich extracts provide anti‐inflammatory and antioxidant activities, lowering the risk of diseases related to inflammation and oxidative stress. In the present study, the TPC of DELF (260.0 ± 0.01 mg GAE/g), together with the identification of methyl gallate (38.15 µg/mg), orientin, isoorientin, ellagic acid, and quinic acid, suggests that these compounds may act synergistically to modulate inflammatory and oxidative pathways. Specifically, methyl gallate has been reported to scavenge free radicals via hydrogen atom transfer [17, 18], while orientin and isoorientin inhibit iNOS expression and reduce NO and pro‐inflammatory cytokine release in microglial cells [25]. Ellagic acid has been shown to suppress NF‐κB and MAPK activation in BV‐2 cells [26], and quinic acid exhibits anti‐neuroinflammatory effects in hippocampal neurons [27]. Thus, based on the promising chemical and nutritional characteristics of DELF, we investigated its anti‐neuroinflammatory activity on lipopolysaccharide (LPS)‐stimulated microglial cells.

Neuroinflammation plays an important role in the onset and progression of neurodegenerative diseases with high social impact, such as Parkinson's and Alzheimer's diseases [28]. In this context, the accumulation and activation of immune cells in the central nervous system (CNS), such as microglia, have a critical role in driving the neuroinflammatory response. Studies have shown that, when activated, microglial cells release various inflammatory mediators, including tumor necrosis factor‐α (TNF‐α), interleukin‐1β (IL‐1β), interleukin‐6 (IL‐6), and nitric oxide (NO). In addition, these cells express receptors for these mediators, which further amplify the inflammatory response. Evidence indicates that this inflammatory environment can influence a host of critical and specialized brain functions, such as neuronal apoptosis, synapse formation, synaptic pruning, and neuronal plasticity [29].

The addition of DELF did not significantly affect cell viability compared to the control group (100% of viability). However, methyl gallate (major constituent) reduced the viability of microglial cells to about 78% at the highest tested concentrations (10 – 20 µg/mL).

**FIGURE 2:**
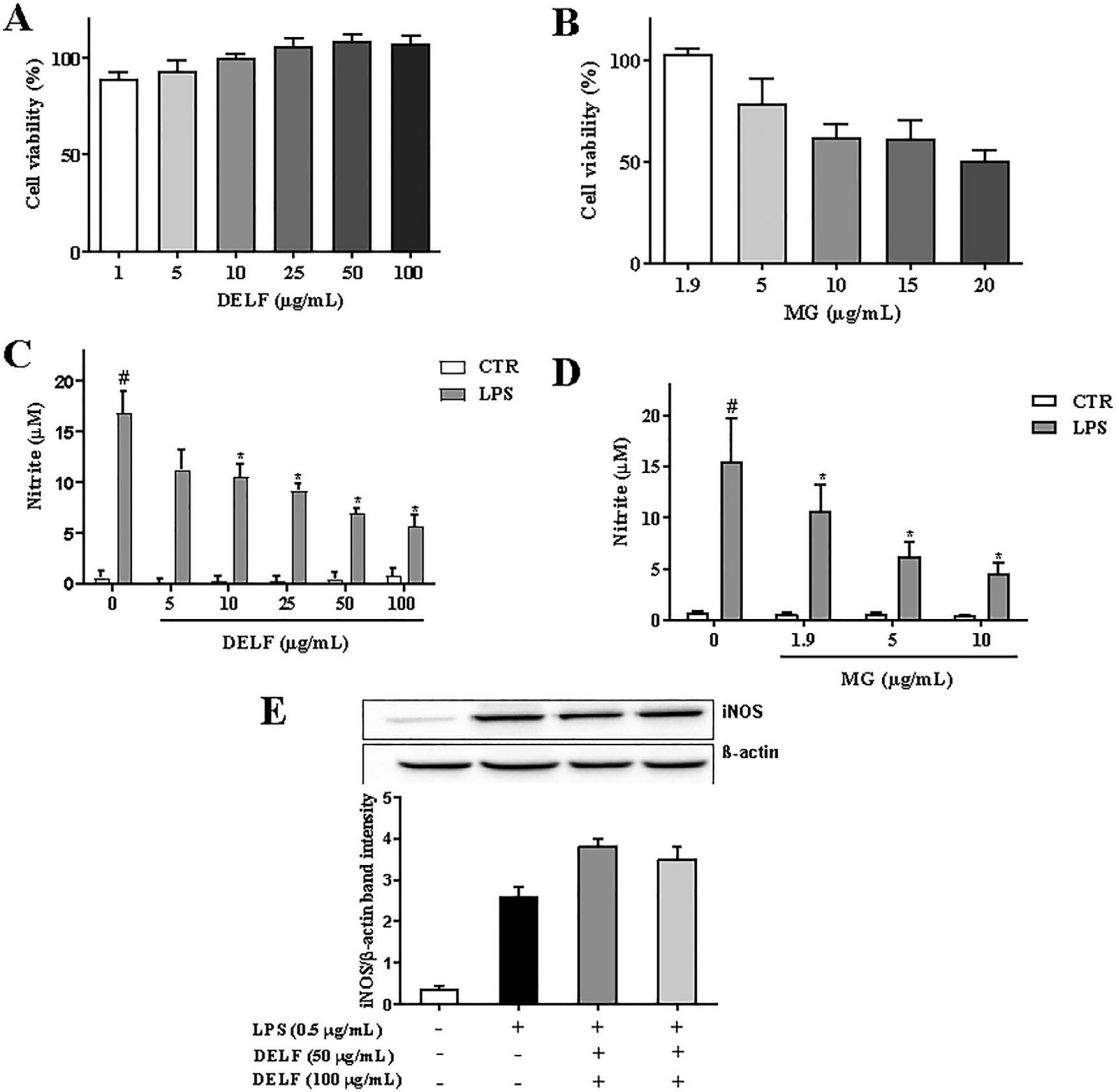
Cytotoxicity by MTT assay of the dry extract from *L. ferrea* (DELF) (A) and methyl gallate (MG) (B), nitrite release of DELF (C) and MG (D), and iNOS expression of DELF in BV‐2 microglial cells. DELF (5, 10, 25, 50, and 100 µg/mL), MG (1.9, 5, 10 µg/mL), vehicle (0.1% DMSO), or DMSO 50% (MTT assay) was added to the BV‐2 cell culture medium. For anti‐inflammatory activity, Lipopolysaccharide (LPS, 0.5 µg/mL) or vehicle (growth medium) was added 1 h later. Griess and MTT tests were performed 24 h later. Bars represent the mean ± SEM. ^#^ *p* < 0.05 versus negative control. *p < 0.05, versus dimethyl sulfoxide (DMSO) 0.1%; two‐way ANOVA followed by Bonferroni post hoc test; *n* = 3/group.

We investigated the role of DELF and methyl gallate in the inhibition of NO, a mediator that is involved in the signaling and development of inflammatory processes, in BV‐2 microglial cells stimulated by LPS, a classic model of neuroinflammation. The addition of DELF or its major constituent, methyl gallate, at increasing concentrations did not alter the basal NO levels in BV‐2 cells (Figure 2C,D). As expected, LPS induced an increase in nitrite release (16.92 ± 3.75 µM); however, pretreatment of the cells with DELF at 10 µg/mL significantly reduced nitrite release into the medium (10.62 ± 2.05 µM) compared to the LPS‐stimulated group. Additionally, a concentration‐dependent reduction in nitrite release was observed with increasing DELF concentrations, with 100 µg/mL resulting in the greatest reduction (5.53 ± 1.91 µM) (Figure 2C). No changes in cell viability were observed following treatment with DELF at concentrations ranging from 1 to 100 µg/mL in the MTT assay (Figure 2A).

The reduction in nitrite release may be related to the presence of methyl gallate. It was observed that a concentration of 1.9 µg/mL (corresponding to the methyl gallate content in 50 µg/mL of the dry extract) significantly reduced nitrite release (11.14 ± 3.87 µM) compared to the LPS‐stimulated control (15.51 ± 6.38 µM) (Figure 2D). Furthermore, a concentration‐dependent tendency for methyl gallate to reduce NO release was observed. However, it should be noted that increasing the concentration above 1.9 µg/mL significantly reduced microglial cell viability (Figure 2B).

Although DELF reduced nitrite release in LPS‐stimulated BV‐2 microglial cells, western blot analysis revealed that this reduction is not associated with a decrease in iNOS expression. The LPS‐stimulated group (iNOS/β‐actin band intensity 2.1 ± 0.07) showed an approximately seven‐fold increase in band intensity compared to the control group (iNOS/β‐actin band intensity 0.3 ± 0.07). However, treatment with DELF at concentrations of 50 and 100 µg/mL did not reduce iNOS protein density when compared to the LPS‐stimulated group (iNOS/β‐actin band intensity 3.2 ± 0.5 and 2.9 ± 0.4, respectively) (Figure 2E).

Notably, the reduction in nitrite release observed in DELF‐treated cells was not accompanied by a decrease in iNOS protein expression, indicating that the observed anti‐neuroinflammatory effect does not rely on transcriptional or translational suppression of iNOS. This finding suggests that DELF may act through post‐translational or non‐enzymatic mechanisms.

Several hypotheses may explain this observation. The phenolic constituents of DELF, particularly methyl gallate and ellagic acid, possess multiple hydroxyl groups capable of directly scavenging reactive nitrogen species, including NO and peroxynitrite, thereby reducing the steady‐state levels of nitrite detected in the culture medium [30]. Additionally, nitrite reduction may occur due to the stimulation of alternative nitrite‐metabolizing pathways, which convert it to products such as nitrate and urea [31, 32].

Recent evidence demonstrates that microglial‐derived NO and reactive oxygen species (ROS/RNS) contribute to neuronal metabolic and oxidative stress, and that pharmacological inhibition of iNOS and NADPH oxidase can attenuate these effects [11]. In this context, the antioxidant properties of DELF demonstrated in the present study (DPPH, superoxide anion, and hydroxyl radical scavenging) may indirectly reduce the formation of peroxynitrite and other secondary reactive nitrogen species, thereby lowering the overall nitrite burden without altering iNOS expression.

Polyphenol‐rich extracts have been shown to modulate microglial polarization and suppress pro‐inflammatory signaling pathways such as NF‐κB, TLR4, and the NLRP3 inflammasome [12], which may contribute to the observed reduction in inflammatory mediators through mechanisms independent of iNOS regulation.

It should be emphasized, however, that these mechanistic interpretations remain hypothetical and require direct experimental validation. Future studies employing specific enzymatic assays (e.g., SOD and catalase activity measurements) are necessary to elucidate the precise mechanisms underlying the anti‐neuroinflammatory effects of DELF.

**FIGURE 3:**
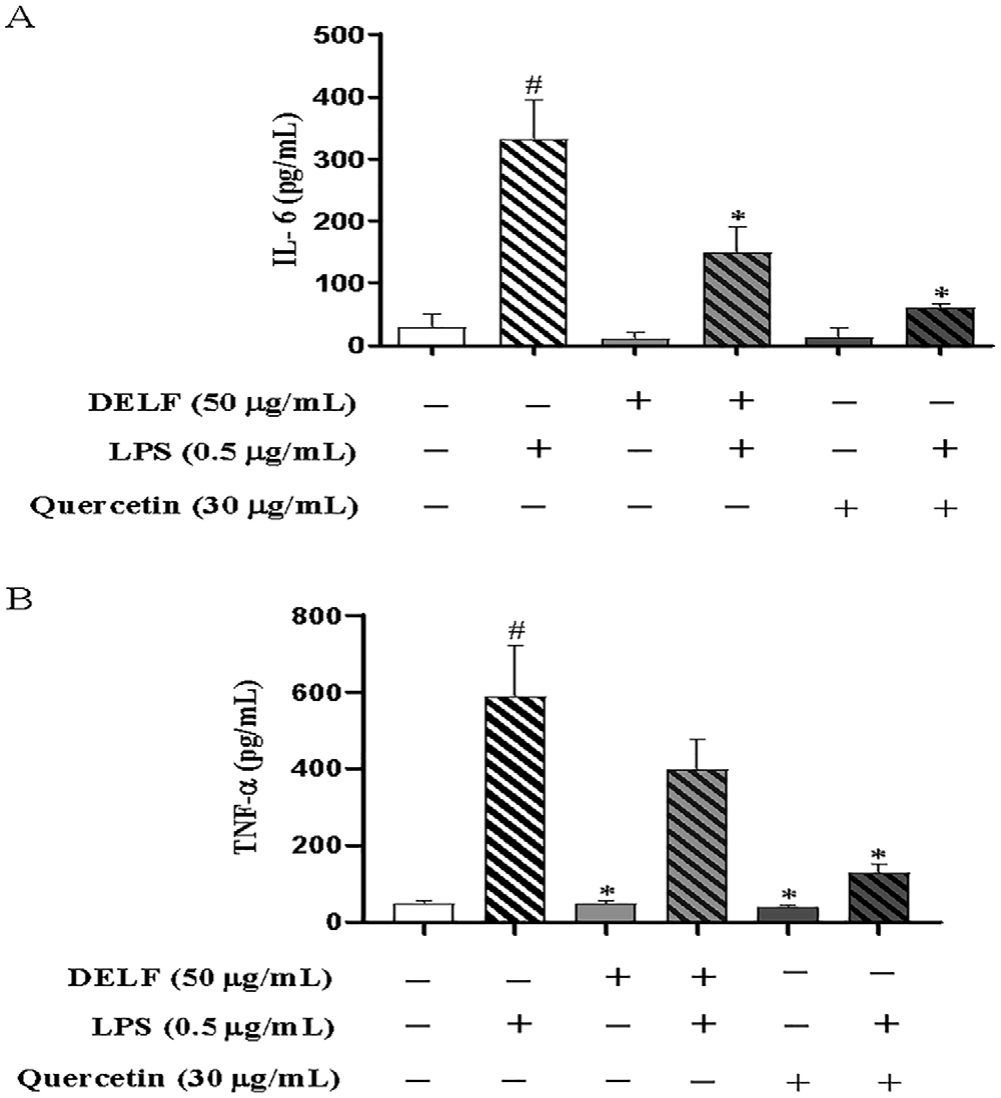
IL‐6 (A) and TNF‐α (B) release of the dry extract from *L. ferrea* (DELF) in BV‐2 microglial cells. DELF (50 µg/mL) or quercetin (30 µg/mL) was added to the BV‐2 cell culture medium. Lipopolysaccharide (LPS, 0.5 µg/mL) was added 1 h later. The levels of IL‐6 and TNF‐α in the culture supernatant were measured 24 h later using ELISA kits. Bars represent the mean ± SEM. ^#^ *p* < 0.05 versus negative control (0.1% DMSO, drug vehicle); *p <0.05 versus LPS group, two‐way ANOVA followed by Bonferroni post hoc test; *n* = 3/group.

Among the effective concentrations tested in the Griess assay, 50 µg/mL was selected for evaluation of its effects on proinflammatory cytokine production in LPS‐stimulated BV‐2 microglial cells. Exposure to LPS for 24 h significantly increased TNF‐α (591.2 ± 132.6 pg/mL) and IL‐6 (333.0 ± 62.5 pg/mL) levels compared with the control group (0.1% DMSO) (50.4 ± 7.3 and 31.1 ± 19.1 pg/mL, respectively). Pretreatment of the cells with DELF for 1 h before LPS stimulation significantly reduced IL‐6 production (150.8 ± 40.6 pg/mL), and this reduction was comparable to that observed with the quercetin standard (61.4 ± 5.6 pg/mL) (Figure 3A). However, TNF‐α production was not significantly reduced compared with LPS‐stimulated control cells (Figure 3B).

The selective reduction of IL‐6 without significant inhibition of TNF‐α production is an intriguing finding that warrants discussion. Although both are pro‐inflammatory cytokines, IL‐6 and TNF‐α are regulated through partially distinct pathways in activated microglia: IL‐6 transcription is strongly dependent on NF‐κB and C/EBPβ activation, whereas TNF‐α is primarily regulated at the post‐transcriptional level through mRNA stability mechanisms [12]. It is possible that the phenolic constituents of DELF preferentially modulate signaling pathways more directly involved in IL‐6 transcription, such as the NF‐κB/IL‐6 axis, without significantly affecting TNF‐α expression.

Additionally, the differential sensitivity may reflect distinct temporal kinetics: TNF‐α is an early‐response cytokine peaking within the first hours of LPS stimulation, whereas IL‐6 exhibits a more sustained expression profile [29]. Since DELF was added 1 h before LPS stimulation, the timing of intervention may have been more effective in attenuating the sustained IL‐6 response rather than the rapid TNF‐α burst. Nevertheless, the lack of TNF‐α inhibition does not diminish the anti‐neuroinflammatory relevance of DELF, as IL‐6 has been specifically implicated in sustaining chronic neuroinflammatory responses and promoting microglial activation in neurodegenerative contexts [11].

The anti‐inflammatory potential of this extract is, in part, associated with the presence of phenolic constituents. A previous study showed that isoorientin inhibits iNOS protein expression, decreases NO production, and inhibits IL‐1β and TNF‐α release (ELISA assay) [25]. Moreover, ellagic acid attenuates the pro‐inflammatory markers NO, TNF‐α, and IL‐1β, increases the release of IL‐10, and suppresses the activation of inflammatory pathways such as mitogen‐activated protein kinase (MAPK), nuclear factor of activated T‐cells (NFAT), and nuclear factor kappa B (NF‐κB) in BV‐2 cells [26].

An in vivo study showed that quinic acid, a polyol found in DELF, reduced neuronal cell death progression in primary rat hippocampal neurons and reduced local neuroinflammation [27]. This data, combined with DELF's ability to modulate microglial activation by reducing the production of inflammatory mediators (such as IL‐6 and nitric oxide) in BV‐2 cells, suggests its potential as an anti‐neuroinflammatory agent. These effects are particularly relevant in contexts where neuroinflammation and oxidative stress may affect the health of the central nervous system.

The variety and synergism of phenolic compounds enable the diversification of endogenous biochemical targets and potentiate the anti‐inflammatory effect. Given the close relationship between oxidative stress and inflammation and the fact that many phenolic compounds also exhibit antioxidant properties, investigating the antioxidant potential of plant extracts is particularly relevant [33]. During periods of redox imbalance, metabolic dysregulation, altered cell signaling pathways, and increased secretion of pro‐inflammatory molecules can potentiate oxidative stress, generating a positive feedback loop. Due to this correlation, antioxidant assessment is of great value for understanding the diseases associated with inflammation [34]. Therefore, we evaluated DELF's antioxidant potential.

Higher intracellular ROS and RNS levels are a result of the organism's inability to clear oxidants and/or higher production of these species, which are related to the pathogenesis of various diseases, including neurodegenerative disorders [35]. The superoxide anion is a precursor of H_2_O_2_ and hydroxyl radicals and is produced in response to several factors, including the activation of immune cells such as neutrophils, macrophages, and microglia. H_2_O_2_ diffuses more easily through the plasma membrane than the superoxide anion, generating higher amounts of hydroxyl radical. However, cells lack enzymatic mechanisms to eliminate hydroxyl radicals [36].

**FIGURE 4:**
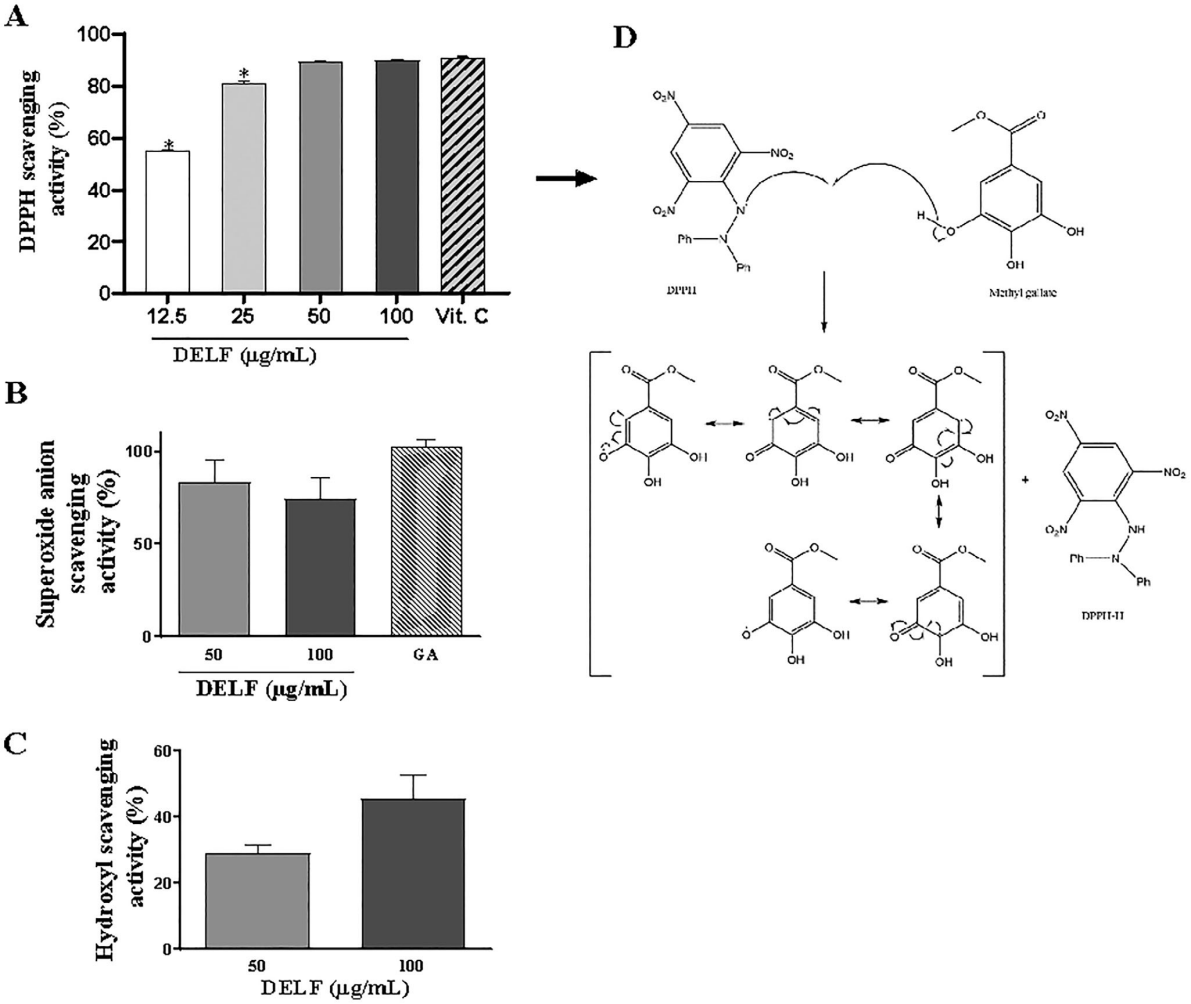
Antioxidant activity of dry extract from *L. ferrea* (DELF): scavenging of DPPH (A), superoxide anion (B), hydroxyl radical (C), and Proposed reaction of DPPH with methyl gallate (DPPH: 1,1‐diphenyl‐2‐picryl‐hydrazyl, DPPH^•^: 1,1‐diphenyl‐2‐picryl‐hydrazyl free radical) (D). Values represent the mean ± SEM; *p < 0.05 versus CTR—or Vit. C; one‐way ANOVA followed by Bonferroni post hoc test; *n* = 3–4/group. CTR, negative control; CTR + positive control GA, gallic acid (100 µg/mL); Vit. C, vitamin C (50 µg/mL).

The antioxidant potential of DELF was evaluated by measuring its scavenging activity on three radical species: DPPH, hydroxyl radical, and superoxide anion (Figure 4). In the DPPH assay, we observed 55.11% scavenging activity of DELF from the low concentrations (12.5 µg/mL), with a maximum effect achieved at a concentration of 100 µg/mL (90.16%). Notably, DELF presented antioxidant activity similar to vitamin C (50 µg/mL) (Figure 4A). This high DPPH radical scavenging activity of DELF is corroborated by Hassan et al. [37], who also demonstrated the antioxidant effect of the aqueous ethanol extract of *L. ferrea*. The authors attributed the scavenging activity to the presence of phenols in these extracts.

We also investigated the ROS‐scavenging effects of DELF on hydroxyl radicals and superoxide anions. The hydroxyl radical is the most reactive and damaging oxidizing species among the free radicals generated in the intracellular environment. It can cause direct damage to vital cellular components, including membrane lipids and the nitrogenous bases of DNA [38]. DELF at 100 µg/mL scavenged approximately 45% of hydroxyl radicals with no significant difference between the 50 and 100 µg/mL concentrations (Figure 4C).

The superoxide anion scavenging activity of DELF was higher than that for the hydroxyl radical (Figure 4B). DELF (50 and 100 µg/mL) showed a maximal superoxide anion scavenging activity of 83.6%. The scavenging activity of DELF is at least partially due to the presence of phenolic acids and flavonoids, such as methyl gallate. This hypothesis is supported by a previous study [17].

The antioxidant properties of DELF are strongly related to the molecular structure of the polyphenols present, that is, the number of hydroxyl and methoxyl groups and their positions on the aromatic ring [18]. We hypothesize that polyphenols such as DELF's major compound, methyl gallate, act by stabilizing free radicals through hydrogen atom transfer, as shown in Figure 4D.

Despite the promising findings, some limitations should be acknowledged. First, the anti‐neuroinflammatory and antioxidant activities of DELF were evaluated exclusively in an in vitro model (LPS‐stimulated BV‐2 microglial cells), requiring validation in in vivo models of neuroinflammation. Although 18 compounds were tentatively identified, only methyl gallate was quantified; while this approach is consistent with established practices for herbal extract standardization, the individual contributions of other identified constituents (e.g., orientin, isoorientin, ellagic acid derivatives, and quinic acid) to the observed bioactivities remain to be fully elucidated. Future studies should also explore the mechanisms underlying NO reduction without iNOS modulation and the selective inhibition of IL‐6 but not TNF‐α.

## 3 Conclusion

This research demonstrates, for the first time, the potential nutritional and anti‐neuroinflammatory properties of the leaf extract of *L. ferrea*, as evidenced by reduced production of the inflammatory mediators nitric oxide and IL‐6 in microglial cells (BV‐2 cell line). In addition, DELF proved to be a source of minerals and phenolic compounds, including methyl gallate and flavonoids. Taken together, the data suggest that DELF could provide benefits as a nutrient‐rich product with anti‐neuroinflammatory potential. From a therapeutic perspective, these findings suggest that DELF may serve as a promising nutraceutical candidate for the prevention or adjuvant treatment of neuroinflammatory conditions. Future studies should focus on in vivo validation, elucidation of the molecular mechanisms underlying NO reduction independent of iNOS modulation, and bioavailability assessments to support potential clinical translation.

## 4 Materials and Methods

### 4.1 Chemicals and Biological Materials

Sodium carbonate, chloric acid, copper sulfate, phosphoric acid, (hydroxymethyl) aminomethane, ethylenediaminetetraacetic acid (EDTA), iron chloride III, sulfonamide, and sodium chloride (NaCl) were purchased from Dinamica, Brazil. Ethanol, dimethyl sulfoxide, quercetin (purity ≥ 98%), N‐(1‐naphthyl)ethylenediamine dihydrochloride (NED), lipopolysaccharide, formic acid, gallic acid (purity ≥ 97%), hypoxanthine, xanthine oxidase, nitroblue tetrazolium, deoxyribose, vitamin C, methyl gallate (purity ≥ 98%), trifluoroacetic acid, thiobarbituric acid, and 3‐[4,5‐dimethylthiazol‐2‐yl]‐2,5‐diphenyltetrazolium (MTT) were obtained from Sigma, USA. The Folin‐Ciocalteau reagent, methanol (HPLC grade), and UPLC‐grade acetonitrile were supplied by Merck (Germany). Laemmli buffer and Polyvinylidene Fluoride membrane (PVDF) membranes were obtained from Bio‐Rad, USA, while aluminum chloride was purchased from Vetec, Brazil, trichloroacetic acid from Exodo, Brazil, and hydrogen peroxide from Synth, Brazil.

Enzyme‐linked immunosorbent assay (ELISA) kits for IL‐6 and TNF‐α were acquired from BD Biosciences Pharmingen, USA. Bovine serum albumin (BSA) and leucine enkephalin were supplied by Waters, USA. The anti‐iNOS antibody was obtained from Abcam, UK. Murine microglial (BV‐2) cells (RRID: CVCL_0182) were acquired from Banco de Células do Rio de Janeiro, Brazil. Reagents for sodium dodecyl sulfate–polyacrylamide gel electrophoresis (SDS‐PAGE), Nonylphenoxypolyethoxyethanol (NP‐40), and Laemmli buffer were acquired from Bio‐Rad (USA). RPMI‐1640 culture medium was obtained from Thermo Fisher, Brazil.

### 4.2 Cell Culture Conditions

The murine BV‐2 cells (RRID: CVCL_0182) were maintained in RPMI‐1640 medium supplemented with 10% fetal bovine serum. The cells were grown to 70%–80% confluence and incubated at 37°C in an atmosphere of 5% CO_2_.

### 4.3 Botanical Materials

The leaves of *L. ferrea* (Mart. ex Tul.) L.P. Queiroz (Fabaceae) was collected in Fortaleza (3°44'45.9'' S, 38°34'39.1'' W), Ceará, Brazil, by Francisco Cirineu das Chagas‐Neto, and the species was identified by the botanist Luiz Wilson Lima‐Verde. The voucher specimen (n° 58169) was deposited in the Herbarium Prisco Bezerra, Department of Biology, Federal University of Ceara, Brazil, and registered in the Brazilian National System of Genetic Resource Management and Associated Traditional Knowledge (SisGen) with registration code A0C462C. The standardized dry extract of *Camellia sinensis* was kindly provided by Givaudan.

### 4.4 Preparation of the Extract of *Libidibia ferrea*

The leaves were crushed and sieved until a moderately coarse powder was obtained. The extract was produced using a combination of extraction methods, turbolysis for 4 min followed by maceration for 30 min. The percentage of the plant drug was 24% in 40% methanol. The extract was rotary evaporated to remove all methanol, then lyophilized, yielding a water‐soluble powder called the dry extract of *L. ferrea* leaves (DELF).

### 4.5 Chemical Characterization of DELF

#### 4.5.1 Total Phenolic Content (TPC)

The TPC in *L. ferrea* leaves extract and in the commercial Camellia sinensis extract were determined using the Folin–Ciocalteu method [39]. One microliter of diluted extracts (1:25 in methanol 40%) was added to 250 µL of Folin–Ciocalteu reagent (Merck) and 3 mL of aqueous sodium carbonate solution (10%) (Dinamica, Brazil). The volume was made up to 10 mL. Samples were vortexed and, after 15 min at room temperature, absorbance was measured at 785 nm using a spectrophotometer (Thermo Scientific Genesys 10S, USA). Results (milligrams of equivalent gallic acid per gram of dry weight) were expressed as mean values subtracted from a blank solution and interpolated in a calibration curve of gallic acid (1‐6 µg/mL). Samples were analyzed in triplicate.

#### 4.5.2 Total Flavonoid Content

The flavonoid content was quantified using a protocol modified from Ahn et al. [40]. In summary, an aliquot of *L. ferrea* extract (1 mg/mL) and a commercial extract of *Camellia sinensis* were added to 800 µL of 2% AlCl3, and the volume was made up to 10 mL. After 20 min at room temperature, the absorbance was measured at 415 nm. Results (mg of equivalent quercetin per gram of dry weight) were expressed as mean values subtracted from a blank solution and interpolated in a calibration curve of quercetin (3‐12 µg/mL). Samples were analyzed in triplicate.

#### 4.5.3 UPLC‐ESI‐QToF‐MS^E^ Analysis

The analysis was performed on an Acquity UPLC (Waters) system coupled to a Quadrupole/Time‐of‐Flight (QTOF, Waters) system. Chromatographic runs were performed on a Waters Acquity UPLC BEH column (15×2.1 mm, 1.7 µm), with a fixed temperature of 40°C. The mobile phase was aqueous 0.1% formic acid (A)—acetonitrile with 0.1% formic acid (B). Samples were eluted using a gradient from 2% to 95% B (9 min); the flow rate was 0.4 mL/min, and the injection volume was 5 µL.

The ESI mode was set to 110–1180 Da, a fixed source temperature of 120°C, a desolvation temperature of 350°C, a desalting gas flow of 500 L/h, an extraction cone of 0.5 V, and a capillary voltage of 2.6 kV. The ESI+ mode was set to 110–1180 Da, a fixed source temperature of 120°C, a desolvation temperature of 350°C, a desolvation gas flow of 500 L/h, and a capillary voltage of 3.2 kV. Leucine enkephalin was the lock mass. The acquisition mode was MS^E^. The compounds were tentatively characterized through molecular formula provided by MassLynx 4.1 software from their accurate masses (error < 5 ppm), isotopic patterns (i‐fit), and MS fragmentation pattern as well as a chemotaxonomic survey. Additionally, compounds were assigned by comparison with available reference standards.

#### 4.5.4 Quantification of Methyl Gallate

Prior to quantification, an aliquot of DELF (25 mg) was subjected to a clean‐up step by solid‐phase extraction (SPE). A C18 SPE cartridge was conditioned with methanol and ultrapure water, and the extract sample was sequentially eluted with 5 mL of water, 5 mL of methanol/water (9:1, v/v), and 5 mL of methanol. The methanol/water (9:1, v/v) fraction was collected, rotary‐evaporated to remove methanol, and lyophilized prior to chromatographic analysis.

The quantification of methyl gallate was carried out by high‐performance liquid chromatography coupled to a diode array detector (HPLC‐DAD) according to the methodology described by Ferreira et al. [41]. Chromatographic separation was achieved on a reversed‐phase C18 column (250 × 4.6 mm), 5 µm particle size; maintained at a fixed temperature of 24°C. The mobile phase consisted of water containing 0.05% (v/v) trifluoroacetic acid (A) and methanol containing 0.1% (v/v) formic acid (B), delivered at a flow rate of 0.8 mL/min. The following gradient elution program was applied: 0–10 min, 12.5%–25% B; 10%–15 min, 25%–40% B; 15%–25 min, 40%–75% B; 25%–30 min, 75% B; 30–33 min, 75%–12.5%. The total run time was 33 min, and the injection volume was 20 µL. Detection was performed with the DAD detector by scanning in the wavelength range of 290–400 nm. Methyl gallate was identified by comparison of its retention time and UV absorption spectrum with those of an authentic reference standard (purity ≥ 98%, Sigma‐Aldrich), and quantification was performed by external standardization. The sample was analyzed in triplicate.

#### 4.5.5 Elemental Determination

The elemental determination was performed using inductively coupled plasma optical emission spectrometry (ICP‐OES) (Perkin Elmer, Model Optima 7000 DV, USA). Six elements (Ca, Fe, Mg, Mn, Pb, and Zn) were quantified using intensity measurements and calibration standards. Samples were analyzed in triplicate, and the data were expressed as ppm, which were then converted to mg per 100 g of dry extract.

### 4.6 Cellular Viability of BV‐2 Microglial Cells (MTT Assay)

The MTT assay is a colorimetric method that measures metabolic activity by converting MTT into formazan crystals [42]. BV‐2 microglial cells were plated at a final density of 1 × 10^5^ cells/mL per well in a 96‐well plate.

Cells were exposed to DELF (1, 5, 10, 25, 50, 100 µg/mL) or methyl gallate (1.9, 5, 10, 15, 20 µg/mL) for 24 h, and then MTT (0.5 mg/mL) was added. Following a 1 h incubation, the cells were centrifuged (130 g, 5 min), and 180 µL of the supernatant was removed; 150 µL of pure DMSO was then added. Absorbance was measured 15 min later at 570 nm. Results were expressed as a percentage of the control group (samples without DELF).

### 4.7 Anti‐Inflammatory Activity on BV‐2 Microglial Cells

#### 4.7.1 Nitrite Determination

The Griess protocol for determining nitrite concentration in the cell medium was performed as described by Green, Tannenbaum, and Goldman [43]. Briefly, DELF (5, 10, 25, 50 and 100 µg/mL) or methyl gallate (1.9, 5, 10 µg/mL) was added to the BV‐2 microglial cell medium (1 × 10^6^ cells/mL) and, 1 h later, LPS (0.5 µg/mL) was added, in a 96‐well plate. 100 µL of culture medium was collected and mixed with 100 µL of Griess reagent. Absorbance was measured at 540 nm and interpolated in a sodium nitrite standard curve to determine the concentration of nitrite in the cell medium. The procedure was performed in triplicate.

#### 4.7.2 The Enzyme‐Linked Immunosorbent (ELISA) Assay

BV‐2 cells were pre‐treated with DELF (50 µg/mL) or 0.1% DMSO (drug vehicle) or quercetin (30 µg/mL) for 1 h, then stimulated with LPS (0.5 µg/mL) for 24 h. The levels of IL‐6 and TNF‐α in the culture supernatant were determined using ELISA kits (BD Bioscience Pharmingen, USA) according to the manufacturer's protocol. The absorbance was measured at 450 nm.

#### 4.7.3 Western Blot Assay

iNOS expression was determined by western blot analysis. Microglial BV‐2 cells (1 × 10^6^ cells/mL) were pretreated with DELF (50 µg/mL), (100 µg/mL), or 0.1% DMSO (drug vehicle) for 1 h. Cells were stimulated with LPS (0.5 µg/mL) for 24 h. Cellular proteins were extracted using radioimmunoprecipitation assay buffer (RIPA), containing protease inhibitor cocktail (1:20), phenylmethylsulfonyl fluoride (1:50), and phosphatase inhibitor cocktail set III (1:20). Protein concentration was defined with the BCA kit using bovine serum albumin (BSA) as a standard and normalized to the same total protein concentration with *Laemmli* buffer. Equal amounts of protein (30 µg) from each sample were subjected to 7.5%–10% SDS‐PAGE and transferred onto polyvinylidene difluoride (PVDF) membranes. After blocking at room temperature with 5% non‐fat dry milk for 1 h, membranes were incubated overnight at 4°C with primary antibodies against iNOS (1:1000, Abcam Cambridge, UK) and β‐actin (1:5000) (Abcam Cambridge, UK). Following three washes with TBS‐T buffer (10 mM Tris‐HCl, pH 7.6; 150 mM NaCl; 0.1% Tween 20), membranes were incubated with the appropriate secondary antibody, anti‐mouse (1:5000) (#170‐6516), for 2 h at room temperature. Protein bands were visualized using an enhanced chemiluminescence reagent (Bio Rad, USA) on an imaging system (Bio Rad ChemiDoc MP), and protein levels were analyzed using Image Lab software v.6.1, and iNOS band intensity was normalized by β‐actin labeling.

### 4.8 Antioxidant Activity

#### 4.8.1 DPPH Radical Scavenging

DPPH radical scavenging activity was measured according to a modified method described by De Gaulejac, Provost, and Vivas [44]. Aliquots (50 µL) of increasing concentrations of DELF (12.5, 25, 50, 100 µg/mL in ultrapure water) were added to 250 µL of DPPH solution (0.05 mg/mL in a methanol/acetate buffer (8:2, v/v, pH 5.4)) in 96‐well plates. The absorbance was measured at 520 nm after 30 min. Vitamin C (50 µg/mL) was used as the standard antioxidant drug. Samples were analyzed in octuplicate. DPPH scavenging percentage was calculated according to the following equation:

$$\%\;DPPH\;scavanging=\frac{\left(ABS\;blank\;-\;ABS\;sample\right)}{\left(ABS\;blank\right)}\times 100$$

*ABS*: absorbance.

#### 4.8.2 Hydroxyl Radical Scavenging

The scavenging activity was measured according to a modified method described by Zhao et al. [45]. First, 20 µL of DELF (50, 100 µg/mL) was added to 20 µL EDTA 1 mM, 20 µL FeCl_3_ 1 mM, 20 µL deoxyribose 36 mM, 20 µl H_2_O_2_ 10 mM, and 20 µL ascorbate 1 mM in 96‐well plates. After 1 h, 200 µL trichloroacetic acid (10%) and 200 µL thiobarbituric acid (1% in NaOH 50 mM) were added and incubated at 85°C for 15 min. The control groups were produced by adding or not adding deoxyribose, an essential substrate that interacts with the hydroxyl radical to form products that, when heated with thiobarbituric acid at low pH, present a pink coloration, as monitored by spectrophotometry in the visible region (532 nm). Substances with antioxidant properties can compete with deoxyribose for the radical, thereby decreasing the intensity of the coloration. Therefore, the percentage of inhibition of the hydroxyl radical was calculated from the comparison between the absorbance values of the samples added with deoxyribose compared to the control with only deoxyribose and total formation of hydroxyl radicals.

#### 4.8.3 Superoxide Anion Scavenging

Superoxide anion production was determined by measuring the enzymatic reduction of nitroblue tetrazolium (NBT) to formazan [46]. DELF (50 µg/mL and 100 µg/mL) was incubated with hypoxanthine (5 mM) and xanthine oxidase (1.67 µg/mL) for 30 min, and absorbance was measured at 650 nm. Gallic acid (100 µg/mL) was used as the standard antioxidant. The amount of residual superoxide anion was calculated using the following equation:

$$\%\;Superoxide\;anion\;residual=\left(P2-P0\right)\;/\;\left(P1\;-P0\right)$$

P_0_ = absorbance of blank; P_1_ = absorbance of positive control for superoxide production; P_2_ = absorbance in the presence of DELF or gallic acid.

### 4.9 Statistical Analysis

All experiments were performed in at least three independent replicates (*n* = 3 per group, unless otherwise stated in the figure legends). Data were checked for normality (Shapiro–Wilk test) and homogeneity of variance (Levene's test); no data transformation or outlier removal was applied. Results are expressed as mean ± SEM. Comparisons among groups were performed using one‐way or two‐way ANOVA (two‐sided, *α* = 0.05), followed by Bonferroni's post hoc test. Statistical analyses were conducted using GraphPad Prism 8.0.1 (GraphPad Software, Inc.), and *p* < 0.05 was considered statistically significant.

## Author Contributions

**Francisco Cirineu das Chagas Neto**: conceptualization, methodology, formal analysis and writing – original draft. **Kirley Marques Canuto**: methodology, validation and visualization. **Arcelina Pacheco Cunha**: methodology, formal analysis and writing – original draft. **Nágila Maria Pontes Silva Ricardo**: methodology, validation and formal analysis. **Ana Bruna de Araújo**: conceptualization, methodology, writing – original draft and writing – review and editing. **Francisco Vinícius Clemente Serra Azul**: conceptualization, methodology and formal analysis. **Paulo R. V. Ribeiro**: conceptualization, methodology, visualization and writing – review and editing. **Patrícia Maria Pontes Thé**: conceptualization, methodology and formal analysis. **Antônia Torres Ávila Pimenta**: methodology, formal analysis and writing – review and editing. **Luzia Kalyne Almeida Moreira Leal**: conceptualization, formal analysis, validation, funding acquisition, visualization, writing – original draft and writing – review and editing.

## Funding

This research was funded by the Coordenação de Aperfeiçoamento de Pessoal de Nível Superior (CAPES), Fundação Cearense de Apoio ao Desenvolvimento Científico e Tecnológico (FUNCAP), and Conselho Nacional de Desenvolvimento Científico e Tecnológico (CNPq).

## Conflicts of Interest

The authors declare no conflicts of interest.

## Data Availability

The data that support the findings of this study are openly available in the Institutional Repository of the Federal University of Ceará at http://www.repositorio.ufc.br/handle/riufc/68905.
